# The burden of Cardiovascular diseases in Jordan: a longitudinal analysis from the global burden of disease study, 1990–2019

**DOI:** 10.1186/s12889-024-18316-0

**Published:** 2024-03-21

**Authors:** Yazan A. Al-Ajlouni, Omar Al Ta’ani, Ghaith Shamaileh, Yazan Nagi, Mohammad Tanashat, Farah Al-Bitar, Dustin T. Duncan, Nour Makarem

**Affiliations:** 1 New York Medical College School of Medicine, 10595 Valhalla, NY USA; 2 Department of Epidemiology, Columbia University Mailman School of Public Health, 10032 New York, NY USA; 3 Allegheny Health Network, 15212 Pittsburgh, PA USA; 4 Tulane University School of Medicine, 70112 New Orleans, LA USA; 5 Faculty of Medicine, Yarmouk University, Irbid, Jordan; 6 Western Michigan University Homer Stryker M.D. School of Medicine, Kalamazoo, MI USA

**Keywords:** Cardiovascular diseases, Jordan, Burden of Disease, Risk factors, Global Burden of diseases

## Abstract

**Background:**

Cardiovascular Disease (CVD) is the leading cause of mortality worldwide. While countries in the Arab world continue to lack public health data and be severely understudied in health research, previous research has shown that compared to 1990, CVDs had a higher burden of disease in the Arab World in 2010. Jordan, a middle-income Arab country, is profiled with unique attributes such as a dual-sector healthcare system, political stability, and its role as a haven for refugees and migrants. These distinctive factors emphasize Jordan’s suitability as a case study. This investigation aims to quantify CVD burden in Jordan and identify risk factors, contributing to a broader understanding of health challenges in the Arab region and beyond.

**Methods:**

The Global Burden of Disease (GBD) dataset was used to estimate prevalence, death, and disability-adjusted life-years (DALYs) as age-standardized rates from 1990 to 2019. We calculated percentage change for nine specific CVDs and reported trends by gender and age groups. Additionally, data on twelve a priori selected behavioral, clinical, and environmental risk factors attributing to overall age-standardized CVDs DALY were reported per 100,00 population.

**Results:**

In 2019, the age-standardized CVD prevalence, death, and DALYs rates in Jordan were 7980 (95% uncertainty interval [UI] 7629, 8360), 248 (95% UI 211, 288), and 4647 (95% UI 4028, 5388), respectively. Despite an increase in the absolute number of mortality and prevalence, between 1990 and 2019, the age-standardized prevalence, death, and DALYs rates all decreased by 5.5%, 45.1%, and 46.7%, respectively. In 2019, the leading risk factors contributing to overall age-standardized CVDs DALY per 100,000 population were high systolic blood pressure, high BMI, dietary risks, and high LDL cholesterol.

**Conclusion:**

Despite decreasing burden rate of CVDs in Jordan between 1990 and 2019, CVDs remain the leading cause of mortality in Jordan, with an increase in the total number of prevalence and mortality. Overall, this contributes to increased healthcare costs. Further research is required to quantify the burden of CVDs and understand it better. Intervention measures and policies tailored to specific CVDs should be designed to reduce the burden of CVDs in Jordan.

## Background

Cardiovascular Diseases (CVDs), which include a number of diseases such as coronary heart disease, cerebrovascular disease, peripheral vascular disease, heart failure, rheumatic heart disease (RHD), congenital heart disease and cardiomyopathies [1], are the leading cause of morbidity and mortality worldwide [2]. It is estimated that approximately one third of deaths worldwide are caused by CVDs [3]. According to the 2017 World Health Organization (WHO) global estimate, each year 17.9 million people die from CVDs, and > 75.0% of these deaths occur in low- and middle-income countries (LMICs) [4]. Among persons aged 30 years without known CVD, the lifetime risk of overall cardiovascular disease approaches 50% [5]. Moreover, underscoring the significance of comprehending the CVD burden and trends, it’s noteworthy that CVD stands as the predominant cause of mortality not only in men and women but also across various socio-economic strata [6, 7].

Globally, the burden of CVD remain the leading cause of disease burden in the world in 2019 [8]. Overall, the literature continues to be lacking on public health data from Arab countries, and the Arab populations in general continue to be understudied in public health research [9]. Analysis of national health accounts of Arab countries has shown that in middle-income Arab countries, government spending, as a percentage of total health expenditure, is decreasing over time [10]. Jordan, a middle-income Arab country, faces key challenges to its health system as reported by The WHO Regional Office for the Eastern Mediterranean [11]. This includes a need for increasing the availability, access, and use of information, as well as increasing research efforts in public health [12]. In the most recent and comprehensive analysis of the burden of diseases in Arab countries, Mokdad et al., demonstrated that ischemic heart disease was the top cause of death in the Arab world in 2010, contributing to 14.3% of deaths [13]. Furthermore, it is notable that Jordan’s Sociodemographic Index (SDI) in 2019 was 0.73, which is higher than the average SDI of other Arab countries in the region, at 0.66 [14]. This disparity in SDI is significant, as it suggests that Jordan might exhibit distinct patterns in CVD prevalence and type compared to the broader region. The higher SDI typically correlates with a transition from infectious diseases to non-communicable diseases like CVD. Compared to 1990, CVDs had a higher burden of disease in the Arab World in 2010. Furthermore, cardiovascular, and other circulatory diseases were the most common causes of death in people aged 40 years or older in middle income countries, including Jordan, in 2010 [13]. However, and to date, there remains a lack of data regarding the epidemiological profile of Arab countries, including Jordan.

Considering Jordan’s distinctive features, including its dual-sector healthcare system (e.g., private and public healthcare), stable political environment, and its role as a haven for refugees and migrants from conflict-ridden neighboring countries, the rationale for selecting Jordan as the focus of investigation becomes evident. Jordan, as a representative Arab Eastern country, is navigating the complexities of a transitioning health landscape. This refers to the dynamic changes in healthcare policies, infrastructure, and response strategies. As Jordan adapts to various health challenges common in the region, such as infectious diseases, non-communicable diseases, resource constraints, and demographic shifts, its experiences hold potential implications not only for its own population but also for other countries within the region facing similar challenges. Despite the significance of this health concern, an evident gap persists in the availability of epidemiological data encompassing Arab countries, including Jordan.

There is a strong need to understand the burden of diseases challenging individual countries, to prioritize the public health system accordingly. Understanding the demographic profile of a disease, as well as its contributing risk factors among a population, can allow public health systems to design policies that address the unique public health needs of a population. Human and financial resources must be allocated strategically to combat diseases, demonstrating a high burden. The Global Burden of Disease (GBD) dataset is an ongoing multinational collaboration to provide comparable and consistent estimates of population health over time [8]. It measures and benchmarks health loss from death or disability from more than 300 diseases in over 100 countries [15]. The GBD dataset provides estimates for deaths, incidence, prevalence, years of life lost (YLLs), years lived with disability (YLDs), and disability-adjusted life-years (DALYs) due to a total of 369 diseases and injuries alongside risk exposure across 87 different risk factors, for both sexes in 204 different countries and territories [16]. The dataset is available publicly using the weblink https://vizhub.healthdata.org/gbd-results/. Further details on the GBD dataset, data sources, processing, and modeling strategies have been published extensively in the literature [8, 16–22].

Upon these bases, this paper sought to investigate trends in the burden of CVD between 1990 and 2019 and to assess the contribution of modifiable risk factors in Jordan using the GBD Dataset. To date, *and to the best of our knowledg*e, the burden of CVDs in Jordan has not been estimated and published in the literature, and there exists no comprehensive evidence regarding the burden of CVDs among the population. Given the very high prevalence of CVDs in Jordan [23], and the burden associated with it, findings from this study can be drastically beneficial in directing public health systems intervention measures and priorities in Jordan.

## Methods

### Data source

This study utilized data obtained from the GBD dataset, an ongoing multinational collaboration to provide comparable and consistent estimates of population health over time [8, 17, 20].

### Measures

#### Cardiovascular diseases

Cardiovascular disease causes are identified with standard case definitions, as detailed below: [8].

1. *Ischemic heart disease (IHD)*: acute myocardial infarction, chronic stable angina, chronic IHD and heart failure due to IHD.
2. *Hypertensive heart disease (HHD)*: symptomatic heart failure due to direct and long-term effects of hypertension.
3. *Non-rheumatic valvular disease (NRVD)*: by its clinical diagnosis.
4. *Stroke*: rapidly developed clinical signs of focal (or global) disturbance of cerebral function, lasting more than 24 h or leading to death, with no apparent cause other than of vascular origin (WHO definition) [24].
5. *Peripheral artery disease (PAD)*: ankle brachial index of < 0.9.
6. *Atrial fibrillation and flutter*: electrocardiogram diagnosis.
7. *Cardiomyopathy (CMY)*: symptomatic heart failure due to primary myocardial disease or toxin exposure to the myocardium.
8. *Acute myocarditis (MYC)*: acute and time limited disease due to myocardial inflammation using health system administrative data.
9. *Endocarditis and rheumatic heart disease (RHD)* were defined by their clinical diagnosis. Estimates of RHD include cases identified by clinical history and physical examination, including auscultation or standard echocardiographic criteria for definite disease.

### Risk factors

The GBD 2019 methods for estimating the burden of disease associated with risk factors has been described in other published work in the literature [8]. The following risk factors were selected from the 2019 GBD data and used in this study: (1) air pollution; (2) alcohol use; (3) dietary risks; (4) environmental/occupational risks (e.g., unsafe water, sanitation, and handwashing; non-optimal temperature, occupational carcinogens, noise, and injures); (5) high body-mass index (BMI); (6) high fasting plasma glucose; (7) high low-density lipoprotein (LDL) cholesterol; (8) high systolic blood pressure; (9) kidney dysfunction; (10) low physical activity; 11) other environmental risks (e.g., residential radon and both acute and chronic exposure to lead); 12) tobacco use. Population-representative survey and surveillance data and geospatial Gaussian process regression models that borrowed strength across time and geography were utilized to estimate risk factor exposures. Detailed descriptions of data sources, metrics and methods are available elsewhere [25].

### Death

Mortality estimates are generated using vital registration data coded to the International Classification of Disease (ICD) system or household mortality surveys known as verbal autopsy.

### DALYs

DALY is the standard metric used to quantify burden [26]. It estimates are the sum of years of life lost due to a specific disability (YLLs) and the years lived with the same disability (YLDs). DALYs were calculated as the sum of YLLs, based on a reference maximum observed life expectancy, and YLDs based on standardized disability weights for each health state [8].

### Prevalence

Prevalence is a measure of the proportion of individuals in a population who have a specific condition or disease at a given point in time. It is often used in epidemiology to describe the burden of a disease within a population. For Jordan, the GBD study uses data from multiple sources such as vital registration data, hospital records, survey data and administrative data. The data is analyzed using a set of statistical models and methods that account for uncertainty and missing data.

### Data analyses

The analysis of GBD data entails a comprehensive approach to data collection, encompassing diverse sources including surveys, medical records, and mortality documentation. Following data acquisition, the GBD study employs advanced techniques such as the Cause of Death Ensemble model (CODEm), spatiotemporal Gaussian process regression (ST-GPR), and DisMod-MR for data processing and adjustment. These methodologies facilitate the delineation of trends in health metrics. A notable tool in this endeavor is the GBD Compare website, which provides a platform for both accessing and interactively visualizing the derived results.

In the context of missing data, the GBD study team adopts a nuanced strategy dependent on the nature of the data gaps. For data characterized by random missingness, the team employs multiple imputation methods to estimate the absent values. Conversely, when data lacks randomness in its missingness, techniques such as inverse probability weighting are applied. Significantly, the team is committed to upholding data quality and methodological transparency, meticulously documenting their approaches to addressing missing data, thus ensuring the integrity of their analytical framework [18, 27]. Further insights into the intricacies of data processing and modeling methodologies can be explored in the referenced scholarly [16–21, 28–30].

## Results

### Prevalence

Table 1 presents all-age and age-standardized prevalence figures for 1990 and 2019. CVD prevalence notably increased from 1990 to 2019 in Jordan, with cases rising from 121,232 in 1990 to 546,588 in 2019. The most substantial relative growth occurred in NRVD (540.0%), followed by CMY and MYC (474.0%) and HHD (425.0%). In 2019, the leading CVD prevalence included IHD (310,388), stroke (134,580), and PAD (71,948). CVD was more prevalent in males in both 1990 and 2019.

**Table 1:** The all-age and age-standardized prevalence number and rate in 1990 and 2019 in Jordan

Subcategory		All-age prevalence number 1990		All-age prevalence number 2019		Change %	Age-standardized rate 1990	Age-standardized rate 2019	change %
All CVD	Male	63,728 (60,329, 67,011)	301,497 (285,705, 318,255)		373.0%		8761 (8364, 9157)	8382 (7984, 8829)	-4.3%
	Female	57,503 (54,440, 60,601)	245,091 (231,389, 258,836)		326.0%		8134 (7776, 8489)	7551 (7185, 7913)	-7.0%
	Total	121,232 (115,308, 127,353)	546,588 (517,925, 574,426)		351.0%		8450 (8104, 8796)	7980 (7629, 8360)	-5.5%
Rheumatic Heart Disease	Male	259 (204, 316)	971 (771, 1221)		275.0%		24 (20, 28)	20 (16, 25)	-16.7%
	Female	329 (269, 396)	978 (781, 1226)		197.0%		31 (26, 37)	23 (19, 28)	-26.0%
	Total	588 (483, 707)	1949 (1579, 2422)		231.0%		27 (23, 32)	21 (17, 26)	-22.2%
Ischemic Heart Disease	Male	38,751 (35,519, 42,377)	196,295 (180,282, 214,002)		407.0%		6227 (5735, 6761)	6066 (5596, 6598)	-2.5%
	Female	24,494 (22,644, 26,429)	114,093 (105,020, 123,577)		366.0%		4219 (3893, 4558)	4060 (3741, 4386)	-3.7%
	Total	63,245 (58,384, 68,586)	310,388 (286,477, 336,917)		391.0%		5232 (4841, 5649)	5112 (4730, 5526)	-2.3%
Stroke	Male	17,126 (15,701, 18,517)	64,714 (57,739, 71,241)		278.0%		2054 (1854, 2240)	1600 (1402, 1795)	-22.1%
	Female	20,421 (18,963, 21,902)	69,867 (64,405, 76,037)		242.0%		2636 (2425, 2829)	2008 (1844, 2184)	-23.8%
	Total	37,547 (34,844, 40,187)	134,580 (123,260, 145,467)		258.0%		2342 (2150, 2519)	1793 (1621, 1952)	-23.4%
Hypertensive Heart Disease	Male	3755 (2691, 5046)	20,585 (14,886, 27,381)		448.0%		628 (456, 858)	657 (477, 879)	4.6%
	Female	2684 (1945, 3593)	13,233 (9701, 17,937)		393.0%		449 (332, 599)	459 (340, 617)	2.2%
	Total	6439 (4678, 8510)	33,818 (25,037, 44,682)		425.0%		536 (395, 710)	561 (417, 747)	4.7%
Non-rheumatic valvular heart disease	Male	375 (281, 493)	2786 (2124, 3470)		643.0%		42 (32, 53)	61 (49, 76)	45.2%
	Female	433 (327, 562)	2387 (1764, 3086)		451.0%		53 (40, 68)	62 (47, 80)	17.0%
	Total	808 (630, 1023)	5173 (4024, 6381)		540.0%		47 (37, 60)	62 (49, 76)	32.0%
Cardiomyopathy and Myocarditis	Male	45 (26, 68)	262 (165, 393)		479.0%		8 (5, 12)	9 (6, 13)	12.5%
	Female	2 (1, 3)	7 (3, 12)		345.0%		0 (0, 0)	0 (0, 0)	0.0%
	Total	47 (27, 70)	269 (172, 398)		474.0%		4 (3, 6)	5 (3, 7)	25.0%
Atrial Fibrillation and flutter	Male	2585 (1975, 3329)	13,880 (10,539, 17,706)		437.0%		467 (356, 603)	473 (362, 598)	1.3%
	Female	2618 (1948, 3419)	12,956 (9757, 16,920)		395.0%		509 (380, 667)	516 (386, 673)	1.4%
	Total	5202 (3941, 6695)	26,836 (20,216, 34,403)		416.0%		490 (372, 634)	494 (379, 633)	0.8%
Peripheral Arterial Disease	Male	6270 (5347, 7221)	33,015 (28,352, 38,167)		427.0%		1041 (894, 1200)	1039 (896, 1190)	-0.1%
	Female	8104 (6907, 9423)	38,933 (33,346, 44,990)		380.0%		1387 (1193, 1613)	1358 (1163, 1563)	-2.1%
	Total	14,374 (12,259, 16,612)	71,948 (61,963, 83,080)		401.0%		1215 (1051, 1401)	1191 (1029, 1367)	-2.0%
Endocarditis	Male	45 (38, 52)	203 (174, 234)		350.0%		4 (3, 5)	4 (4, 5)	0.0%
	Female	45 (38, 52)	191 (161, 225)		325.0%		5 (4, 6)	5 (4, 6)	0.0%
	Total	90 (77, 104)	394 (337, 455)		337.0%		4 (4, 5)	5 (4, 5)	25.0%
Other CVD and circulatory disease	Male	4700 (3498, 6316)	17,823 (13,543, 23,367)		279.0%		289 (228, 370)	304 (240, 387)	5.2%
	Female	6925 (5066, 9186)	28,434 (20,954, 37,910)		311.0%		540 (404, 718)	561 (423, 747)	3.9%
	Total	11,624 (8671, 15,352)	46,257 (35,078, 60,258)		298.0%		408 (312, 529)	418 (325, 539)	2.5%

**Abbreviations: CVD = Cardiovascular Disease**

The age-standardized prevalence of CVD per 100,000 population decreased 5.5% from 8450 in 1990 to 7980 in 2019. Notable reductions occurred for RHD (-22.2%) and stroke (-23.4%). Conversely, NRVD saw a significant increase (32.0%), as did CMY and MYC (25%) and endocarditis (25.0%). Leading the prevalence rates in 2019 were IHD (5,112 per 100,000), stroke (1,793 per 100,000), and PAD (1,191 per 100,000). Furthermore, the age-standardized prevalence rate of IHD was higher for males in 2019 (6066) than for females (4060), while stroke prevalence was higher for females (2008) compared to males (1600).

### Mortality

Table 2 presents all-age and age-standardized death figures for 1990 and 2019. Over this period, annual deaths attributed to CVD increased, rising from 4,534 in 1990 to 11,798 in 2019. Among specific subcategories, PAD saw the most substantial relative growth in deaths (486.0%), followed by atrial fibrillation and flutter (358.0%), and HHD (230.0%). Notably, the overall CVD mortality rate per 100,000 population decreased by 45.1%, declining from 451 in 1990 to 248 in 2019. Leading causes of death in 2019, as per CVD age-standardized rates, included IHD, stroke, and HHD.

**Table 2:** The all-age and age-standardized death number and rate in 1990 and 2019 in Jordan

Subcategory		All-age Death number 1990	All-age Death number 2019	Change %	Age-standardized rate 1990	Age-standardized rate 2019	change %
All CVD	Male	2184 (1812, 2538)	6683 (5371, 8297)	206.1%	402 (335, 466)	249 (200, 306)	-38.1%
	Female	2350 (2015, 2677)	5115 (4221, 6212)	117.6%	499 (425, 565)	249 (206, 299)	-50.1%
	Total	4534 (3972, 5111)	11,798 (10,089, 13,862)	160.2%	451 (394, 507)	248 (211, 288)	-45.1%
Rheumatic Heart Disease	Male	10 (6, 14)	14 (10, 20)	46.0%	1 (0, 1)	0 (0, 0)	-100.0%
	Female	16 (10, 20)	15 (12, 21)	-2.0%	1 (1, 2)	0 (0, 0)	-100.0%
	Total	25 (19, 32)	29 (23, 36)	16.0%	1 (1, 2)	0 (0, 0)	-100.0%
Ischemic Heart Disease	Male	1318 (1121, 1543)	3883 (3098, 4871)	194.6%	226 (193, 264)	135 (108, 167)	-40.2%
	Female	1049 (892, 1214)	2228 (1807, 2709)	112.4%	222 (190, 256)	107 (87, 129)	-51.8%
	Total	2367 (2076, 2692)	6111 (5196, 7286)	158.2%	225 (197, 254)	121 (103, 144)	-46.2%
Stroke	Male	592 (440, 717)	1673 (1207, 2120)	183.0%	122 (90, 147)	69 (49, 86)	-43.4%
	Female	834 (670, 986)	1694 (1349, 2069)	103.0%	179 (142, 212)	83 (66, 100)	-53.6%
	Total	1426 (1185, 1662)	3367 (2758, 3983)	136.2%	150 (123, 175)	75 (61, 89)	-50.0%
Hypertensive Heart Disease	Male	182 (132, 231)	840 (490, 1092)	361.1%	37 (27, 47)	34 (20, 44)	-8.1%
	Female	357 (265, 496)	940 (595, 1188)	163.2%	78 (56, 111)	46 (28, 58)	-41.0%
	Total	540 (423, 680)	1780 (1119, 2140)	230.0%	58 (45, 74)	39 (24, 47)	-32.8%
Non-rheumatic valvular heart disease	Male	6 (4, 8)	22 (16, 28)	256.0%	0 (0, 1)	0 (0, 0)	0.0%
	Female	8 (6, 11)	18 (14, 24)	114.0%	1 (1)	0 (0, 0)	0.0%
	Total	15 (12, 18)	40 (33, 48)	174.0%	1 (0, 1)	0 (0, 0)	0.0%
Cardiomyopathy and Myocarditis	Male	13 (7, 18)	30 (21, 42)	134.0%	1 (0, 2)	0 (0, 1)	-100.0%
	Female	9 (5, 13)	13 (10, 20)	48.0%	1 (0, 1)	0 (0, 0)	-100.0%
	Total	22 (13, 29)	43 (33, 59)	98.0%	1 (0, 1)	0 (0, 0)	-100.0%
Atrial Fibrillation and flutter	Male	13 (8, 16)	63 (43, 80)	405.0%	3 (2, 4)	3 (2, 4)	0.0%
	Female	18 (14, 22)	76 (59, 93)	325.0%	5 (3, 6)	5 (3, 6)	0.0%
	Total	30 (24, 37)	139 (113, 163)	358.0%	4 (3, 5)	4 (3, 5)	0.0%
Peripheral Arterial Disease	Male	0 (0, 0)	3 (2, 4)	618.0%	0 (0, 0)	0 (0, 0)	0.0%
	Female	0 (0, 0)	2 (1, 2)	345.0%	0 (0, 0)	0 (0, 0)	0.0%
	Total	1 (1)	4 (3, 5)	486.0%	0 (0, 0)	0 (0, 0)	0.0%
Endocarditis	Male	9 (6, 11)	25 (18, 34)	185.0%	0 (0, 1)	0 (0, 0)	0.0%
	Female	13 (7, 18)	30 (20, 40)	123.0%	1 (1, 2)	1 (0, 1)	0.0%
	Total	22 (15, 28)	54 (42, 69)	147.0%	1 (1)	0 (0, 1)	-100.0%
Other CVD and circulatory disease	Male	26 (21, 32)	75 (57, 95)	186.0%	4 (3, 5)	2 (2, 3)	-50.0%
	Female	40 (32, 52)	80 (64, 104)	102.0%	6 (5, 9)	3 (2, 4)	-50.0%
	Total	66 (56, 81)	155 (128, 186)	135.0%	5 (4, 7)	2 (2, 3)	-60.0%

Abbreviations: CVD = Cardiovascular Disease

In 2019, no discernible difference existed in the overall mortality rate per 100,000 between males and females. However, gender-specific differences in mortality rates were noted for specific causes. Notably, men exhibited a higher mortality rate for IHD, while women demonstrated a higher mortality rate for stroke, HHD, atrial fibrillation and flutter, and endocarditis.

### DALYs

Table 3 presents all-age and age-standardized DALY figures for 1990 and 2019. Age-standardized CVD DALYs decreased by 46.7% between 1990 and 2019, declining from 8724 to 4647, respectively. Notably, a greater overall reduction in CVD DALYs was observed among women (-52.6%) compared to men (-40.5%). The most significant percentage reduction in age standardized DALYs was observed for RHD (-74%), followed by CMY and MYC (-52.6%), stroke (-50.2%), IHD (-48.3%), endocarditis (-46.2%), NRVD (-42.3%), HHD (-34.1%), PAD (-14.2%), and atrial fibrillation and flutter (-3.4%). A greater decline of age-standardized CVD DALYs was observed among women across all CVD subcategories.

**Table 3:** The all-age and age standardized DALYs number and rate in 1990 and 2019 in Jordan

Subcategory		All-age DALYs number 1990	All-age DALYs number 2019	change %	Age-standardized rate 1990	Age-standardized rate 2019	change %
All CVD	Male	60,290 (50,704, 70,128)	174,484 (141,022, 214,801)	189.0%	8383 (7049, 9698)	4984 (4048, 6104)	-40.5%
	Female	55,109 (47,707, 62,704)	115,814 (97,598, 139,375)	110.2%	8988 (7778, 10,189)	4258 (3588, 5086)	-52.6%
	Total	115,399 (101,998, 130,044)	290,299 (251,499, 340,796)	151.6%	8724 (7708, 9798)	4647 (4028, 5388)	-46.7%
Rheumatic Heart Disease	Male	383 (257, 548)	534 (392, 718)	39.3%	35 (23, 50)	11 (8, 15)	-68.6%
	Female	653 (434, 854)	574 (438, 774)	-12.0%	59 (39, 76)	14 (10, 18)	-76.3%
	Total	1036 (798, 1293)	1108 (900, 1348)	7.0%	46 (35, 58)	12 (10, 15)	-74.0%
Ischemic Heart Disease	Male	36,510 (30,693, 43,227)	102,393 (81,332, 127,821)	180.5%	4875 (4162, 5710)	2764 (2211, 3442)	-43.3%
	Female	22,598 (18,982, 26,497)	45,088 (36,552, 55,577)	99.5%	3816 (3245, 4429)	1694 (1389, 2057)	-55.6%
	Total	59,108 (51,111, 67,885)	147,481 (124,436, 177,498)	149.5%	4382 (3842, 4982)	2265 (1931, 2687)	-48.3%
Stroke	Male	15,826 (12,715, 18,688)	42,996 (33,734, 52,810)	171.7%	2420 (1823, 2868)	1341 (1037, 1637)	-44.6%
	Female	21,010 (17,666, 24,490)	43,122 (35,716, 50,905)	105.2%	3397 (2813, 3941)	1567 (1301, 1843)	-53.9%
	Total	36,836 (31,690, 42,514)	86,118 (72,852, 100,150)	133.8%	2910 (2466, 3357)	1448 (1220, 1679)	-50.2%
Hypertensive Heart Disease	Male	4346 (3163, 5461)	18,920 (11,898, 24,442)	335.3%	707 (524, 891)	615 (376, 783)	-13.0%
	Female	7336 (5468, 9831)	18,499 (12,684, 23,344)	152.2%	1304 (969, 1793)	720 (470, 903)	-44.8%
	Total	11,682 (9439, 14,257)	37,419 (25,921, 45,440)	220.3%	1008 (800, 1254)	664 (440, 797)	-34.1%
Non-rheumatic valvular heart disease	Male	200 (139, 265)	660 (486, 858)	230.0%	22 (15, 28)	15 (11, 20)	-31.8%
	Female	262 (191, 343)	524 (407, 685)	100.0%	31 (23, 39)	14 (11, 19)	-54.8%
	Total	462 (365, 575)	1183 (972, 1447)	156.2%	26 (21, 32)	15 (12, 18)	-42.3%
Cardiomyopathy and Myocarditis	Male	576 (310, 827)	1157 (838, 1526)	100.8%	43 (24, 61)	24 (18, 33)	-44.2%
	Female	414 (205, 592)	513 (385, 734)	23.9%	31 (17, 43)	12 (9, 17)	-61.3%
	Total	991 (575, 1345)	1671 (1319, 2180)	68.7%	38 (23, 50)	18 (15, 25)	-52.6%
Atrial Fibrillation and flutter	Male	388 (297, 499)	1966 (1485, 2562)	406.2%	78 (60, 100)	76 (58, 96)	-2.6%
	Female	457 (357, 572)	2067 (1605, 2605)	352.6%	98 (77, 121)	94 (74, 116)	-4.1%
	Total	845 (666, 1068)	4033 (3169, 5063)	377.2%	88 (70, 110)	85 (68, 104)	-3.4%
Peripheral Arterial Disease	Male	35 (20, 58)	176 (105, 284)	400.0%	6 (4, 11)	6 (3, 10)	0.0%
	Female	41 (22, 70)	172 (91, 293)	319.0%	8 (4, 13)	7 (3, 12)	-12.5%
	Total	76 (41, 127)	348 (196, 578)	356.0%	7 (4, 12)	6 (3, 11)	-14.2%
Endocarditis	Male	374 (235, 502)	920 (637, 1245)	145.8%	29 (20, 38)	18 (13, 25)	-38.0%
	Female	491 (253, 671)	961 (601, 1284)	95.9%	50 (27, 68)	24 (16, 33)	-52.0%
	Total	865 (548, 1122)	1881 (1376, 2360)	117.5%	39 (26, 50)	21 (16, 26)	-46.2%
Other CVD and circulatory disease	Male	1167 (946, 1444)	3138 (2507, 3904)	168.8%	105 (85, 129)	69 (55, 86)	-34.3%
	Female	1694 (1385, 2040)	3823 (2972, 4877)	125.7%	170 (140, 213)	94 (75, 117)	-44.7%
	Total	2862 (2420, 3405)	6961 (5700, 8489)	143.3%	137 (116, 166)	80 (67, 95)	-41.6%

**Fig. 1:**
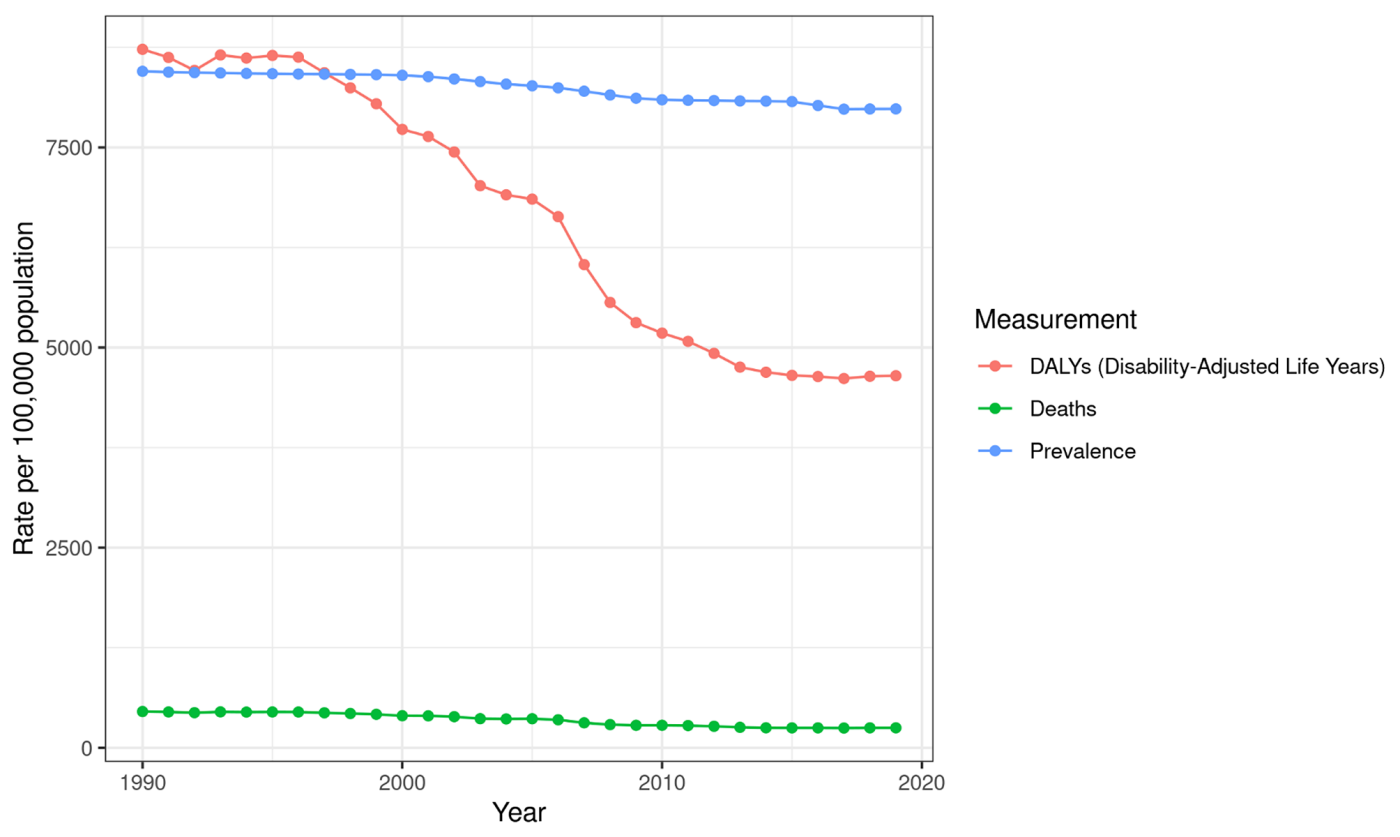
CVDs burden in Jordan: Age standardized DALYs, deaths, and prevalence rates per 100,000 for both genders from 1990–2019

Figure 1 visualizes CVDs burden in Jordan by plotting age standardized DALYs, deaths, and prevalence rates per 100,000 for both genders from 1990 to 2019.

### Risk factors for CVD

**Fig. 2:**
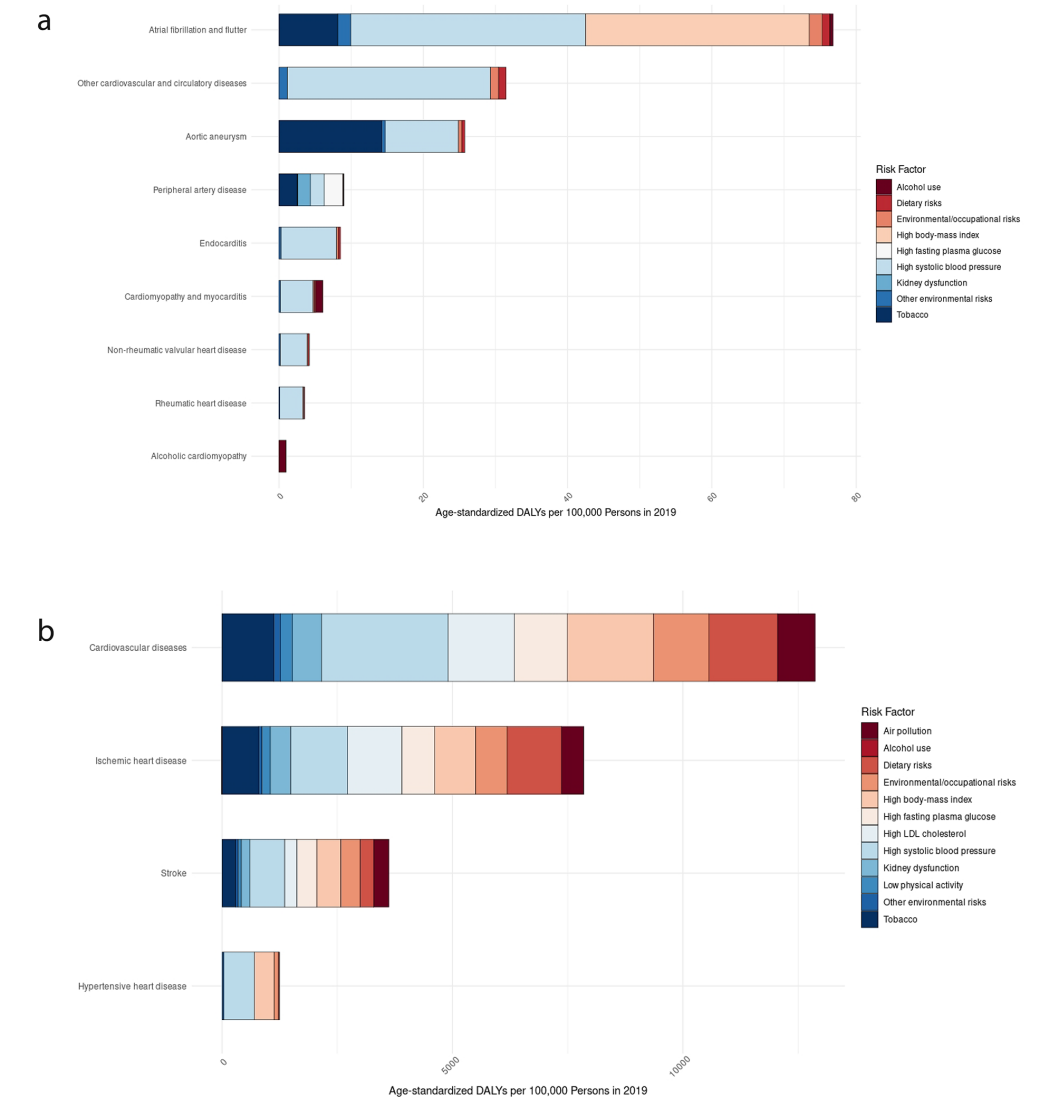
**A**. Age standardized cardiovascular disease (CVD) DALYs per 100,000 persons attributable to risk factors in 2019; **B**. Age standardized cardiovascular disease (CVD) DALYs per 100,000 persons attributable to risk factors in 2019* 2i2 * Note: The data presented in this figure includes 12 risk factors for the highlighted categories. However, for other categories, a subset of 9 relevant risk factors were available in the dataset, as shown in Fig. 2A. The distinction in the number of risk factors included contributes to the variations observed in the depicted trends

Figure 2 illustrates the age-standardized CVD DALYs per 100,000 persons attributed to risk factors in 2019. Based on the GBD dataset, the DALYs rate is associated with 12 risk factors across CVD subcategories. In 2019, the leading risk factors contributing to overall age-standardized CVD DALYs per 100,000 population were high systolic blood pressure, high BMI, dietary risks, and high LDL cholesterol. Risk factors displayed considerable variation across CVD subcategories.

For the top three prevalent CVD cases—IHD, stroke, and PAD—leading risk factors included dietary risks, high systolic blood pressure, and high fasting plasma glucose, respectively. High systolic blood pressure emerged as the primary risk factor for DALYs associated with endocarditis, CMY and MYC, NRVD, RHD, and other cardiovascular and circulatory diseases. Additionally, it was a prominent contributor to DALYs for IHD, stroke, and HHD.

High systolic blood pressure and high BMI predominantly attributed to DALYs for atrial fibrillation and flutter, while smoking surfaced as the primary risk factor for aortic aneurysm. High systolic blood pressure remained a significant contributor to DALYs for IHD, stroke, and HHD.

### CVD by sex

Tables 1, 2 and 3 show the findings of this paper by gender. Overall age-adjusted prevalence was higher among males compared to females. Between 1990 and 2019, the absolute number of CVD cases among males increased from 63,728 to 301,497, and among females, it increased from 57,503 to 245,091. Using the age-standardized metric, CVD prevalence was also higher for males (8,382) than females (7,551) in 2019. There was no difference in sex for age-standardized mortality rate. However, age standardized DALYs were higher in males compared to females, with values of 4,984 and 4,258, respectively.

### CVD by age groups

**Fig. 3:**
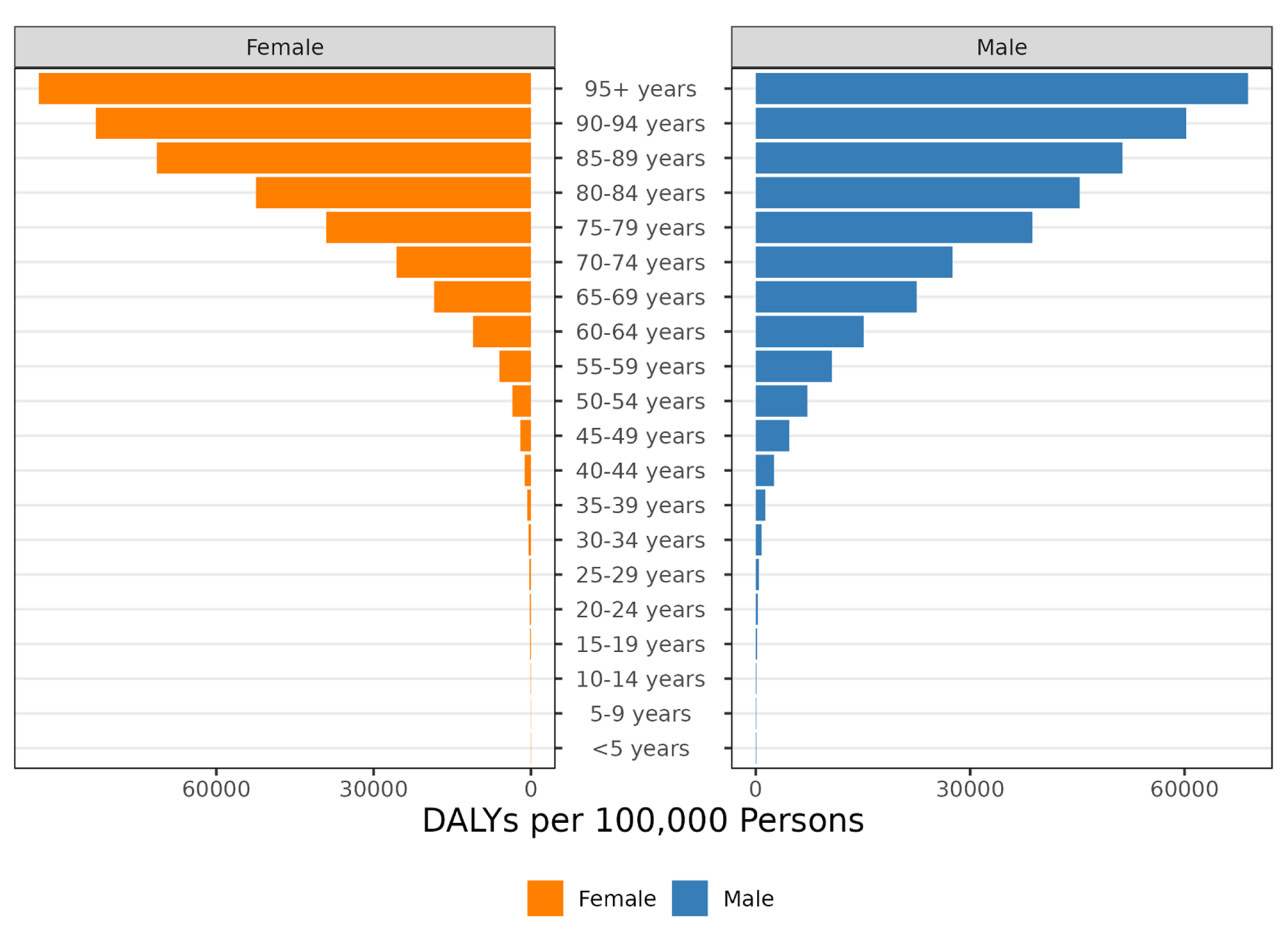
Gender-Specific Burden of Cardiovascular Disease by Age Group in Jordan

Figure 3 shows the DALYs rate across all age groups for both genders, males vs. females. The burden of CVD by exhibits distinct patterns across different age groups and between genders. The analysis reveals that DALY rates per 100,000 persons for both males and females increase with age. However, the burden among males is consistently higher across nearly all age groups. The DALY rates for males show a gradual increase with the advancing age, reaching the highest burden in the 95 + year age group. In contrast, females experience a rise in DALYs beginning in the 65–69-year age group, with a peak observed in the 85–89-year category, followed by a slight decrease in rates for those aged 90–94 years and 95 + years. Furthermore, the disparity between genders is most notable in the post-65 age groups, where male DALY rates surpass those of females significantly, suggesting a higher age-related burden of CVD in males. In younger age brackets, specifically those below 50 years, the gender differences in DALY rates are less marked. This pattern shifts in older age groups, where the gap in DALY rates between males and females becomes more pronounced.

## Discussion

Jordan is under-studied within the context of public health research, and its distinctive socio-political and healthcare system characteristics render it an intriguing setting to study. Additionally, this underscores the need for comprehensive investigations into critical health issues in the country. To the best of our knowledge, this is the *very first study* to report the burden of total and individual CVDs in Jordan and to highlight leading contributing risk factors to the growing CVD burden using the GBD dataset. Overall, there was a decrease in age-standardized prevalence rates, mortality rates, and DALYs rates for CVD from 1990 to 2019. Interestingly, there was a sex difference in the burden (e.g., age standardized DALYs rate) of CVD, with females having a more drastic decrease (52.6%) in comparison to males (40.5%). In terms of burden of specific CVD diseases, HD, CMY, and MYC, and stroke witnessed the greatest decrease in burden rates among the Jordanian population between 1990 and 2019 (-74.0%, -52.6%, and − 50.2%, respectively). On the other hand, atrial fibrillation and flutter had the lowest rate of decrease in their disease burden during that period (-3.4%), which was also concurrent with atrial fibrillation and flutter accounting for the second largest relative growth in the number of deaths (358.0%).

Additionally, while the number of deaths increased, the age-adjusted mortality rates attributed to CVDs decreased drastically during that period. In sum, despite public health gains related to reductions in age-adjusted CVD prevalence and morality rates, the number of cases and deaths is increasing, and the CVD burden remains significant in the Jordanian population. In Jordan, CVD deaths have been reported to be the main cause of death, where 1 in 4 deaths is attributed to CVDs [31]. Although the literature is lacking on this topic among the Jordanian population, our findings are consistent with those reported by countries neighboring Jordan with similar epidemiological and behavioral profiles, as well as with larger epidemiological studies conducted in the region. For instance, it has been reported that the burden of CVDs decreased from 1990 to 2015 in all Eastern Mediterranean Region (EMR). Jordan had the third most drastic decrease in burden during that time, following Bahrain and Qatar [31]. This decrease in burden witnessed in Jordan may be attributed to many factors, including behavioral modifications and medical interventions. The WHO Global Status Report on noncommunicable diseases demonstrated that Jordanians have the highest level of physical activity among EMR countries [32]. Furthermore, the WHO STEPwise study, which is a household based, interviewer-administered survey, demonstrated that Jordanians had the highest medication rate for dyslipidemia (62.1%) among countries in the EMR [33]. Similarly, Jordanians were shown to have the highest medication rate for hypertension (83.7%) [34].

Furthermore, our findings highlight a greater prevalence of CVDs among males compared to females, consistent with extensive literature examining global gender-related disparities in CVD burden. This pattern is evident not only within our study but also across neighboring countries like Saudi Arabia [35]. Men’s propensity to develop CVD at an earlier age than women has been well-established [36], and this risk escalates more rapidly in men as they age [37]. Equally vital is the recognition that women often contend with more severe CVD risk factors, even more pronounced within Arab populations [38]. The interplay of biological, lifestyle, and societal factors likely contributes to these gender differences in CVD burden.

Moreover, gender-specific behaviors play a pivotal role. Men’s inclination toward riskier habits such as alcohol consumption, drug use, and smoking, which are established CVD risk factors, must be acknowledged. This is further compounded by the societal burden disproportionately borne by women, a phenomenon linked to caregiver stress which has implications for CVD risk. Pregnancy-related complications also emerge as a CVD risk factor among women, despite limited data within the MENA region. Nevertheless, it’s crucial to recognize that beyond the data, women face unique stressors stemming from social norms, biases, and discrimination—factors amplifying CVD risk and its downstream impact [39, 40]. The dynamic interplay of these factors underscores the imperative for targeted strategies in both prevention and intervention. This underscores the importance of tailored medical interventions, which meticulously consider gender-specific risk factors, while also addressing the societal and lifestyle components that collectively contribute to gender disparities in CVD burden. By adopting such a holistic approach, we not only accommodate biological distinctions but also navigate the broader sociocultural context, fostering effective and equitable CVD care pathways.

Building on this, our findings on the age-related burden of CVD in Jordan, particularly among males, align with global epidemiological evidence indicating a rise in CVD prevalence and mortality with age. The observed increase in DALY rates with advancing age, as reported in our study, reflects patterns noted in cross-sectional and demographic-specific studies [41–43]. These patterns are not isolated but are echoed by the GBD 2015 and the CASSANDRA model, which underscore the importance of age in understanding the CVD epidemiology and projecting disease burden [44, 45]. Moreover, our data correspond with findings from diverse population studies, such as those conducted on geriatric populations in India and on postmenopausal women, further substantiating age as a pivotal determinant of CVD burden [46, 47]. The convergence of these findings with our own emphasizes the necessity of integrating age and gender-specific considerations into medical interventions. By adopting such a holistic approach that accounts for both biological differences and the wider sociocultural landscape, more effective and equitable pathways for CVD care can be developed, addressing not only the individual risk factors but also the collective societal influences that contribute to the observed disparities in CVD burden.

The high burden of cardiovascular disease in Jordan is a complex issue with multiple contributing factors, and there remains opportunities for tailored interventions despite reports in the literature of high physical activity and medication rates among Jordanians [23]. For instance, high systolic blood pressure and high LDL cholesterol were shown to be significant contributors to the burden of CVDs in general. The high prevalence of those risk factors may be due to the limited knowledge and awareness of CVDs and their risk factors among Jordanians, which has been previously established in the literature [48–53]. For example, among Jordanian patients with acute coronary syndrome, an estimated mean delay of 7.8 h from symptoms onset to coronary reperfusion was attributed to a lack of awareness about symptoms, resulting in a delay in seeking medical care [48]. Other studies in the literature also reported lack of knowledge regarding healthy behaviors and the necessity for controlling risk factors among Jordanian CVDs patients [49–53]. These findings regarding risk factors of CVDs highlight the need for comprehensive strategies to address the underlying causes of cardiovascular disease in the country.

### Public health implications

The findings presented in this study underscore the critical public health implications of CVDs. The study unveils disparities in disease burden trends among different CVDs, with conditions like atrial fibrillation and flutter showing minimal improvement, signaling the need for targeted interventions. Additionally, general public health interventions are needed to reduce the prevalence of CVDs risk factors in the Jordan. Addressing underlying diseases as risk factors (e.g., high systolic blood pressure, high LDL cholesterol, high blood glucose levels) may be done through educational programs aimed at informing the public. Eshah et al., reported the effectiveness of an education, counseling and behavioral skill-building program in Jordanian working adults’ knowledge, attitudes, and beliefs about CVDs and adoption of a healthy lifestyle [54]. Among other populations, educational programs addressing behaviors were shown to impact subjects’ bio-physiological indicators, including significant reductions in body weight, waist circumference, serum lipids, glucose level, blood pressure, and resting heart rate [55–62]. Additional interventions can include policy initiatives to promote healthy diets and increase physical activity, as well as efforts to reduce tobacco use and improve access to healthcare services. During the COVID-19 pandemic, Saifan et al., demonstrated the effectiveness of telehealth services in enhancing the accessibility of healthcare provision among CVD patients [63]. By addressing some of the issues highlighted in our study that are contributing to the burden of CVDs in Jordan, it may be possible to reduce the burden of CVDs in the Jordan and improve the cardiovascular health of the population.

### Strengths and limitations

This paper is not without noteworthy limitations, which are inherent to the data source. The GBD dataset is a valuable tool for tracking the impact of diseases and injuries on populations around the world. Despite this, the dataset has several limitations that should be considered when interpreting the results. The GBD dataset relies heavily on data from vital registration systems, which may be incomplete or of poor quality in many countries, especially in developing countries with less advanced public health systems. Particularly, death certificates may misclassify causes of death, causing an underestimation of CVD cases. This is mainly because access to CVD diagnostic technologies is likely to influence estimates, with lower access resulting in lower observed prevalence. In addition, the GBD dataset may not accurately capture the burden of certain diseases and injuries, particularly those that are difficult to diagnose or are not routinely reported to health authorities.

Furthermore, another noteworthy limitation of this investigation is the minimal availability of data on regional epidemiology, hindering the ability to make comprehensive comparisons and validate the obtained results against regional benchmarks. The absence of such contextual information may limit the generalizability of findings beyond the national level. Moreover, the lack of specific data on the SDI within Jordan poses a challenge in analyzing the results within a broader socio-economic context. The absence of this crucial information prevents a more nuanced understanding of the interplay between disease burden and socio-economic factors, potentially limiting the depth of interpretation and policy implications. Moreover, the reliability of data related to Jordan may be compromised, particularly in rural areas where surveillance systems may be less robust. The potential under-representation of these regions in the dataset raises concerns about missing or incomplete data, which could impact the accuracy of prevalence estimates. Finally, this study recognizes a limitation in data comparability due to the substantial increase in the immigrant population in Jordan by 2019, not present in the 1990 dataset, potentially impacting the homogeneity and direct comparability of the data over the study period. Despite this, this article presents the burden of CVD as the first national estimate for Jordan and underlines the need to establish a national system to estimate national burden and causes of death due to CVD.

### Future research

Future research should incorporate enhanced data collection and analysis to capture the incidence, severity, and disability caused by CVD, while also investigating co-morbidities and the combined effects of multiple risk factors. In light of the COVID-19 pandemic, there is a critical need to assess the pandemic’s impact on CVD burden in the MENA region, potentially uncovering interactions between CVD and COVID-19 and guiding strategies for managing both conditions. To effectively address the root causes of CVD, future studies should prioritize investigating the prevalence and control of traditional risk factors such as hypertension and diabetes. A comprehensive understanding of the influence of lifestyle and dietary factors on cardiovascular health is essential, necessitating research on the impact of diet, physical activity, and other modifiable behaviors. Evaluating interventions designed to enhance access to healthy food options and increase physical activity levels is pivotal, encompassing the assessment of community-based education programs and healthcare policies. Ultimately, bridging the gap between characterizing the burden of CVD, identifying optimal solutions, and ensuring equitable delivery to the entire population is imperative.

## Conclusion

From 1990 to 2019, there was a decrease in age-standardized prevalence rates, mortality rates, and burden rates for CVD. Interestingly, a more drastic decrease in the burden of CVD was observed among females (52.6%) in comparison to males (40.5%). In terms of burden of specific CVD diseases, HD, CMY, and MYC, and stroke witnessed the greatest decrease in burden rates among the Jordanian population between 1990 and 2019. Despite the decrease in overall burden, CVDs continue to be the leading cause of mortality among Jordanians, with increasing overall mortality numbers and healthcare costs. Future research should continue to quantify the burden of CVDs in Jordan and design interventions to further decrease this burden.

## Data Availability

All data generated or analyzed during this study are included in this published article.

## Abbreviations

CVD: Cardiovascular Disease
RHD: Rheumatic Heart Disease
WHO: World Health Organization
LMICs: Low and Middle-Income Countries
YLL: Years of Life Lost
YLD: Years Lived with Disability
DALY: Disability-Adjusted-Life Years
GBD: Global Burden of Disease
EMR: East Mediterranean Region
IHD: Ischemic Heart Disease
HHD: Hypertensive Heart Disease
NRVD: Non-Rheumatic Valvular Disease
PAD: Peripheral Artery Disease
CMY: Cardiomyopathy
MYC: Acute Myocarditis
ICD: International Classification of Disease
LDL: Low-Density Lipoprotein
